# Is Autism a Member of a Family of Diseases Resulting from Genetic/Cultural Mismatches? Implications for Treatment and Prevention

**DOI:** 10.1155/2012/910946

**Published:** 2012-06-26

**Authors:** Staci D. Bilbo, John P. Jones, William Parker

**Affiliations:** ^1^Systems and Integrative Neuroscience Group, Department of Psychology and Neuroscience, Duke University, Durham, NC 27710, USA; ^2^Department of Surgery, Duke University Medical Center, Durham, NC 27710, USA

## Abstract

Several lines of evidence support the view that autism is a typical member of a large family of immune-related, noninfectious, chronic diseases associated with postindustrial society. This family of diseases includes a wide range of inflammatory, allergic, and autoimmune diseases and results from consequences of genetic/culture mismatches which profoundly destabilize the immune system. Principle among these consequences is depletion of important components, particularly helminths, from the ecosystem of the human body, the human biome. Autism shares a wide range of features in common with this family of diseases, including the contribution of genetics/epigenetics, the identification of disease-inducing triggers, the apparent role of immunity in pathogenesis, high prevalence, complex etiologies and manifestations, and potentially some aspects of epidemiology. Fortunately, using available resources and technology, modern medicine has the potential to effectively reconstitute the human biome, thus treating or even avoiding altogether the consequences of genetic/cultural mismatches which underpin this entire family of disease. Thus, if indeed autism is an epidemic of postindustrial society associated with immune hypersensitivity, we can expect that the disease is readily preventable.

## 1. Introduction: Autism as a Member of a Large Family of Postindustrial Epidemics Involving a Hyperimmune Response

 In this paper, we outline a paradigm that points toward autism as one disease among many other well-known diseases which all share a common origin and, most likely, a common prevention strategy [1]. These emerging, noninfectious diseases are all epidemics of postindustrial culture, which are absent in antiquity in any culture, and absent in present day developing cultures. Members of this family of disease are invariably associated with a hyperimmune response and can have a very high prevalence in postindustrial populations, with prevalence often greater than 0.1% and sometimes greater than 1.0%. These hyperimmune-associated diseases include a wide range of allergic, autoimmune, and inflammatory diseases such as lupus, multiple sclerosis, hay fever, appendicitis, chronic fatigue syndrome, inflammatory bowel disease, asthma, celiac disease, type 1 diabetes, Graves' disease, some types of eczema, and a wide range of food allergies. Here, based on a variety of evidence, we argue that autism is yet another member of this family of diseases, despite the slowness of the medical community to recognize it as such. Since the pathogenesis of at least one form of autism has been directly linked to autoantibody production [2], and since seven out of every eight cases of severe autism are associated with antineuronal antibodies in the serum [3], this idea has already been demonstrated, at least in part. Further, as described below, autism is associated with a wide range of immune abnormalities. Even though some children with autism appear to have “normal” immune systems, it is argued that adverse immune events early in development might lead to an autistic phenotype.

 A primary consideration in this view of autism as a hyperimmune-associated disease is the connection between immunity and brain development in general, and between hyperimmune responses and autism in particular. Although these connections have little bearing on the overall model describing induction of disease by immune hypersensitivity in postindustrial society, the connections provide reason to expect that the developing brain is sensitive to the same postindustrial changes in the immune system which are known to affect virtually all other organs of the human body (e.g., kidneys, pancreas, epidermis, adult nervous tissues, large and small bowel, cardiovascular system, thyroid, lungs, and others). The next four sections will summarize much of what is currently known regarding those very extensive connections.

## 2. Pervasive Immune System Abnormalities in Autism

Immune system abnormalities exist throughout the body and brain of autistic children. These include evidence of brain specific auto-antibodies, altered T, B, and NK cell responses to antigen, altered cytokine production, an increased incidence of allergies and other autoimmune disorders, and functional changes in brain glial cells (microglia and astrocytes) [4–11]. Microglia are the primary immunocompetent cells of the brain and rapidly respond to any infection, injury or other perturbation of homeostasis via a dynamic process of activation [12]. Notably, increased microglial activation has been observed in several brain regions of autistic patients [13]. Once activated, glia produce a wide number of immune signaling molecules, including cytokines (e.g., interleukin [IL]-1*β*, tumor necrosis factor [TNF]-*α*), chemokines (e.g., monocyte chemoattractant protein [MCP]-1), and other inducible factors (e.g., nitric oxide), which may profoundly influence neural function [14].

## 3. Immune System-Central Nervous System Communication

 Beyond its traditional role in host defense and tissue repair, the immune system is now considered a diffuse sensory organ that works in concert with the endocrine, metabolic, and nervous systems to achieve and maintain homeostasis throughout the body [15, 16]. In essence, the immune system serves as an interface between the human body and the environment, coordinating the response of the body to the environment. Immunocompetent cells are located throughout every organ of the body, including the brain, and regular communication occurs between the central nervous system and immune tissues during both health and disease processes. Many excellent reviews have been written on these topics [17–20]. Importantly, bidirectional communication between the brain and immune system has significant consequences for plasticity mechanisms within the brain, including cognition and emotion, which are markedly altered in autism.

## 4. Glial Cells Direct Normal Brain Development

Microglia are the resident macrophages of the central nervous system, are associated with the pathogenesis of many inflammatory diseases of the brain, and derive from primitive yolk sac macrophage precursors, which are of mesodermal origin and enter the neuroectoderm during embryogenesis [21]. Early in development, microglia are highly mobile and primarily amoeboid, consistent with their role in the phagocytosis of apoptotic cells [22]. The expression of many cytokines within the developing brain, including IL-1*β* and TGF-*β*, depends on the presence of amoeboid microglia [23]. Microglia transform into a highly branched, ramified morphology by adulthood in most brain regions. This morphological transition occurs in parallel with neural cell genesis and migration, synaptogenesis, and synaptic pruning, suggesting functions for microglia in each of these processes, though these are just beginning to be explored [24–26]. Oligodendrocytes myelinate axons primarily during the postnatal period, and disruption of their function can be profoundly debilitating as in the case of periventricular leukomalacia leading to cerebral palsy [27]. Astrocytes mediate synapse formation within the developing brain [28], in part via the secretion of extracellular matrix proteins called thrombospondins (TSPs) [29, 30]. Alterations in spine density via a putative TSP mechanism are implicated in neurodevelopmental disorders such as Down's and Rett Syndrome [31]. Notably, astrocyte maturation marks the end of the perinatal synaptogenic period when the brain is most plastic [32].

## 5. Long-Term Consequences of Immune Activation during Early Development

Developmental or “fetal programming” is a growing field of science based on evidence that experiences during critical or sensitive periods of perinatal life may modulate or “program” the normal course of development, with the result that adult outcomes, including behavior, are significantly and often permanently altered [33]. Given the many ways in which the immune system is important for normal brain development, the capacity for immune-inducing events to influence the long-term trajectory and function of these processes is likely profound, perhaps more so than at any other stage of life [34]. Notably, there is strong evidence that early-life infection can permanently alter stress reactivity, disease susceptibility, and notably, vulnerability to cognitive and neuropsychiatric disorders, including Alzheimer's disease, Parkinson's disease, schizophrenia, and autism [35–38]. One of the most dramatic and direct illustrations of this effect is the induction of autism-like symptoms in mice by stimulation of the maternal immune system during pregnancy [39].

 Neuroinflammatory-induced alterations in neurogenesis, migration, axon growth, myelination, synaptogenesis, and dendritic spine density/function are all candidate mechanisms by which long-term changes in behavior might result. Further, the long lifespan of microglia and their pattern of central nervous system colonization during development suggest that these immune cells may have particular significance in terms of neuroinflammatory effects on developmental programming. Diverse early-life events may have enduring consequences for the brain and behavior of organisms via an influence on these resident immune cells, both by impacting their own intrinsic function as well as their interactions with ongoing developmental processes [40, 41].

## 6. Approaching Autism as Another Member of the Family of Hyperimmune-Associated Diseases: Addressing the Underlying Causes

The study of individual postindustrial associated diseases has often failed to enlighten researchers as to the underlying cause of diseases. While genetic factors associated with the epidemics of postindustrial society abound, these genetic factors have not changed over many generations, and thus are not responsible for newly emerging epidemics of disease. Almost paradoxically, even when specific triggers are identified, these often cannot be said to cause newly emerging epidemics. For example, peanuts and ragweed pollen are two well-known triggers for allergy, but these substances have been present in the environment for millennia without triggering disease in humans. Similarly, viral infections and potentially some milk proteins [42] have been identified as triggers for particular autoimmune diseases, but these factors have been present for much longer than the diseases they trigger. Thus, as will be described in detail, it is not the genetics of the disease which point toward a cure, and it is generally not identification of the triggers for disease that is most helpful. Rather, we suggest that the most effective and insightful approach toward defeating autism for future generations will be the neutralization of underlying factors in postindustrial culture which render postindustrial humans uniquely susceptible to disease, despite the presence of the same genetics and many of the same triggers as found in preindustrial cultures. These factors have been identified as mismatches between normal human genetics and the environment of postindustrial culture. With that in mind, an examination of the factors in postindustrial culture which trigger the plagues of hyperimmune-associated diseases will be presented.

## 7. Mismatches between Normal Human Genetics and Postindustrial Culture Leading to Hyperimmune Epidemics: Biome Depletion

**Figure 1:**
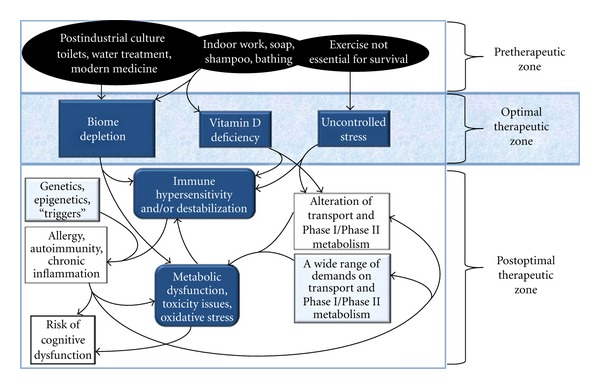
The pathogenesis of epidemics of allergic, inflammatory, and autoimmune disease. Cultural mismatches with human biology (the pretherapeutic zone) have consequences that can be readily avoided (the optimal therapeutic zone). If biome depletion in particular is not avoided, the individual is left susceptible to a wide range of often complex immune-related diseases (the postoptimal therapeutic zone), sometimes associated with imbalances in metabolite transport and/or Phase I/Phase II metabolism. Other consequences such as vitamin D deficiency and uncontrolled stress may, in some but not all cases, add to the problem. Factors contributing to disease which are often naturally occurring and generally difficult or impossible to avoid are underlined and fit within the postoptimal therapeutic zone.

If indeed autism fits into the family of hyperimmune-associated diseases plaguing postindustrial culture, then the pathogenesis or origins of these diseases merit detailed analysis in any consideration of autism. As shown in Figure 1, postindustrial culture has several mismatches with normal human genetics (known as “evolutionary mismatches”) which have a profoundly destabilizing effect on the human immune system. This effect is closely intertwined with an impact on metabolism and transport, as will be described later. These mismatches of postmodern culture involve some of the most cherished attributes of postmodern culture, including the widespread use of toilets, water treatment facilities, indoor working environments, soaps and shampoos, modern medicine, and the loss of factors which demand exercise for survival (e.g., human-eating predators, hardship in finding food and procuring shelter for most of the population). These mismatches are not a target for therapy, because reversal of any of these mismatches is untenable. Thus, these mismatches can be described as belonging to the “pretherapeutic zone” in the course of pathogenesis (Figure 1). However, these mismatches lead directly to readily measurable consequences that, in turn, lead to disease if left untended. Fortunately, these direct consequences of mismatches are readily treated or even avoided altogether. Given that these consequences can be treated or avoided, this part of the pathogenesis of hyperimmune disease can be described as the “optimal therapeutic zone” (Figure 1). In this paper we describe how mismatches between culture and genetics/biology have led to specific consequences which cause immune system hypersensitivity and/or destabilization. All of these consequences are associated with a wide range of diseases that include allergic, autoimmune, and inflammatory diseases.

The single consequence of genetic/culture mismatches that most profoundly impacts hyperimmune-associated disease has been described as a depletion of components from the natural ecosystem of the human body, or simply “biome depletion” (Figure 1). The use of modern medicine, toilets, water treatment facilities, and cleaning agents in our culture has led to a substantial alteration or even loss of living organisms that have coevolved with humans. Three primary components of the biome which are potentially important have been affected [43]: (a) loss of interactions with soil bacteria, (b) alterations of the microbiome, the normal bacteria that are associated with the human body, and (c) loss of helminths, worms that typically inhabit the gut of mammalian species. Substantial evidence points toward all three of these factors as being important in stabilization of the human immune system. Although not widely appreciated, several lines of evidence point very strongly if not conclusively to the idea that helminths in particular are necessary for a stable immune system. This evidence has been reviewed extensively [1, 43] and can be very briefly summarized as follows.

- Helminths are found almost ubiquitously in preindustrial humans [44] and in nondomesticated mammals, including chimpanzees [45]. Even when helminths cannot be found, the immune system shows signs of stimulation by helminths [46].
- Helminths produce and secrete a number of molecules which regulate the host immune system [47].
- Addition of helminths to laboratory animals blocks allergic, inflammatory, and autoimmune diseases [47].
- Addition of helminths to humans cures inflammatory bowel disease [48] and stops the progression of multiple sclerosis [49].
- The effects of biome depletion on the immune system are not necessarily instantaneous because long-term effects of immune interactions with the biome can span decades or, through epigenetics, even generations [1]. Nevertheless, the epidemiology of hyperimmune disease reveals an inverse correlation between helminths and disease.
- Humans with helminths and rodents colonized with helminths have profoundly less reactive immune systems than do either without helminths [44, 50, 51].
- A long coevolutionary history of helminths and vertebrates, in conjunction with the potent immunoregulatory effects of the former, is consistent with the idea that the human immune system is literally, physically dependent on helminths [1].

We propose that biome depletion is changing the immune system at a population-wide level, which in turn will undoubtedly impact the brain, particularly during sensitive periods of development. Importantly, this model does not compete with any prior hypotheses or known risk factors for neuroinflammatory conditions, but rather complements them. For instance, biome depletion may set the stage for exaggerated levels of neuroinflammation, which interacts with other known risk factors or triggers (psychological or metabolic stress, infection, genetics or epigenetics) in the induction of autism. 

## 8. Mismatches between Normal Human Genetics and Post-Industrial Culture Leading to Hyperimmune Epidemics: Vitamin D Deficiency

Based on the available information outlined above, it seems very likely that biome depletion is by far the most significant and most profoundly influential consequence of genetic/cultural mismatches in postindustrial society. However, other consequences which apparently exacerbate the effects of biome depletion are evident. For example, vitamin D deficiency has reached epidemic proportions in postindustrial society, is known to impact inflammation during early development [52], and, like biome depletion, has been linked to a spectrum of allergic, autoimmune, and inflammatory diseases [53–55]. Further, epidemiology and other circumstantial evidence link vitamin D deficiency to autism [56, 57]. Interestingly, the widely publicized association between rainfall and autism [58] can be accounted for, perhaps more than in part, by decreased exposure to sunshine in areas with high rainfall, and the subsequent impact of lower vitamin D levels.

Vitamin D deficiency is another consequence of postindustrial culture's mismatches with human biology (Figure 1). Vitamin D production requires exposure of oils on the body's surface to sunlight. The resulting photochemical reaction and production of vitamin D is greatly reduced by components of postindustrial culture that reduce exposure to sunlight (e.g., indoor work environments, sunscreen).

The association of vitamin D deficiency with a spectrum of hyperimmune-associated diseases places this consequence of biology/culture mismatch alongside biome depletion as an underlying agent destabilizing immune system function in postindustrial populations. However, vitamin D deficiency is an ancient problem, as indicated by the ancient occurrence of rickets, whereas epidemics of hyperimmune-associated diseases are more recent in nature. Thus, it seems likely that vitamin D deficiency is a contributor to hyperimmune-associated disease, but not sufficient by itself to lead to disease. Further, the prevalence of vitamin D deficiency in postindustrial society is somewhat less than the prevalence of biome depletion: although the prevalence of vitamin D deficiency is substantial, with one study finding 24% of individuals deficient (≤20 ng/mL) with an additional 34% having levels that were insufficient (≤29 ng/mL) [59], the prevalence of biome depletion in postindustrial populations is essentially 100%. Thus, it is expected that the impact of vitamin D deficiency on immunity in postmodern culture may be a matter of exacerbating the problems associated with biome depletion more so than a significant problem when considered in isolation.

Some mechanisms by which vitamin D might stabilize the immune system have been elucidated. Vitamin D acts as a signaling molecule to enhance immune cell function in the presence of infection and is important for maintaining the normal interface between the microbiome and the immune system [60]. However, the mechanisms by which vitamin D interacts within the body are complex, with the molecule being intertwined either directly or indirectly in virtually all aspects of human biology. The active form of vitamin D binds and activates the Vitamin D receptor, altering the expression of a variety of genes involved in such diverse processes as cell growth and proliferation, bone remodeling, and calcium homeostasis [61]. It has been estimated that vitamin D binds human DNA in more than 2,500 places and changes the expression of more than 200 genes [62]. Thus, while it is known that vitamin D is required for normal immune function, it is likely that vitamin D deficiency could cause a general destabilization of the human biome independent of the immune system, particularly during early development. Fortunately, regardless of the extent to which vitamin D deficiency destabilizes the human biome, dietary supplements are readily available, and thus this consequence of culture/biology mismatch is easily avoided.

## 9. Mismatches between Normal Human Genetics and Postindustrial Culture Leading to Hyperimmune Epidemics: Other Factors

 The above discussion points out two factors, primarily biome depletion and secondarily vitamin D deficiency, which generally destabilize the human immune system. These factors can be identified by their association with a wide range of hyperimmune-associated diseases. Other consequences of postindustrial culture can also be found which are associated with a wide range of disease, but limits in space prevent a complete discussion. For example, chronic uncontrollable psychosocial stress is yet another factor that is generally associated with a range of hyperimmune-associated diseases that include allergy, autoimmunity, and inflammation [63–66]. Such stress derives from a complex range of biology/culture mismatches, including, for example, the loss of physical exercise [67–69] as a requirement for survival in postindustrial culture (Figure 1). As another example of consequences of biology/culture mismatches which destabilizes immune function, deprivation from mother's milk during infancy (not included in Figure 1) is associated with a wide range of allergic and autoimmune conditions [70–74]. This genetic/culture mismatch, like other mismatches, involves an aspect of postindustrial society which should not be reversed (the survival of infants despite the unavailability of mother's milk), and thus fits into the “pretherapeutic zone” of Figure 1. The extent to which factors such as uncontrollable stress and lack of mother's milk can contribute to disease in the presence of an otherwise normal biome remains to be elucidated, although, as outlined above, biome depletion appears to be the single most important factor contributing to the increased incidence of hyperimmune-associated diseases.

## 10. Metabolism and Transport of Toxins and Hyperimmune-Associated Disease

The connection between metabolism and immunity is important in a consideration of hyperimmune-associated diseases. A variety of enzymes have been identified which are useful in the biotransformation of endogenous (produced by the body or the associated biome) as well as exogenous (pharmaceutical) substrates. This system of enzymes provides the body with a means of processing a wide range of substances, some of which are toxins, and is profoundly affected by the immune system [75]. The metabolic portion of this system is readily divided into two components: Phase I biotransformation includes the oxidative reactions of the microsomal cytochrome P450 monooxygenase system that is expressed in the liver and gastrointestinal tract, and to some extent in a variety of extrahepatic tissues. Phase II biotransformation includes conjugation reactions of substrates with endogenous cofactors such as glucuronate, sulphate, acetate, or glycine.

In addition to Phase I and Phase II biotransformation, the transport of their substrates and byproducts is also an important component of this system. A variety of membrane spanning polypeptides have been identified in the liver, gastrointestinal tract, kidney as well as other tissues in the body, each responsible for the active trafficking of substrates across cell membranes. These transport proteins are in fact regulated by the same pathways that regulate the Phase I and II enzymes, and they can be thought of as being regulated in tandem [76–80].

Immune activation results in a downregulation of Phase I components [81, 82], transporters [83, 84] and potentially Phase II components in what might be viewed as a tradeoff between immunity and metabolism. In time of duress, when the immune system is geared to inflammation and defense, the energy devoted to metabolism/transport may need to be temporarily diverted to host defense. This is apparently an effective strategy, but as a consequence, chronic immune activation could result in metabolic difficulties as outlined in Figure 1. Unfortunately, vitamin D deficiency is expected to make this problem worse, since vitamin D is required to upregulate both Phase I and Phase II components [85] of metabolism. Further, accumulation of toxins as a result of reduced Phase I, Phase II, or transport activity may also stimulate the immune system, adding further to the propensity for hyperimmune activity.

## 11. Implications of Autism as a Member of the Family of Hyperimmune-Associated, Postindustrial Diseases

 The implications of this model are several-fold and deserve careful consideration. The consideration of some possible assertions that are *not* supported by this model is probably equally as important.

- This model indicates that prophylactic normalization of the biome will result in a profound decrease in the incidence of autism. Results with other hyperimmune-associated diseases have been promising, although a true normalization of the biome as a means of prevention has never been attempted for any disease.
- This model is consistent with the idea that a variety of factors affecting immunity and/or metabolism/transport may strongly influence the incidence of autism.
- There are several potential therapeutic approaches that this model does *not* suggest which will necessarily be successful. For example, this model does not predict that helminths will necessarily work as a cure for autism. Whether helminths can be used to treat autism will depend on the extent to which autism is reversible and the extent to which ongoing problems with biome stability can be reversed.
- Although porcine whipworms are currently being used to treat the effects of biome depletion in some studies, the model described in this paper does not predict that the porcine whipworm will prevent autism, even prophylactically. (Although the use of porcine whipworms is currently underway for a few clinical studies, the use of porcine whipworms as a means of prophylaxis is probably not economically feasible because the costs of giving the necessary biweekly doses would be prohibitive. This problem is not expected when using helminths adapted to humans, since one dose lasts for years.) It might, but this potentially depends on the extent to which this species recapitulates the presence of a normal biome. Thus, if porcine whipworms do not work, it does not suggest that the hypothesis is wrong.
- This model does not suggest that helminths are the only organisms involved in biome depletion. Normalization of the microbiome may also be a critical factor. In the face of modern practices in obstetrics and of widespread antibiotic use, this is an issue that must be considered.
- Pharmaceuticals may never prove adequate. The complexity of the immune system and the effects of biome depletion suggest that efforts to readjust the system using drugs may be naive and overly optimistic. Biome reconstitution may be the easiest and most straight forward way to bring the system into homeostasis. Similarly, metabolic factors may not easily be compensated for by pharmaceuticals. Normalization and regulation rather than compensation and suppression are probably the better approaches.
- Genetics may be relatively unimportant from a clinical perspective because it may not provide a means to prevent or treat disease. Triggers may also be unimportant from a clinical perspective because (a) they may simply be unavoidable, and (b) even if triggers for a particular disease can be identified and avoided, such efforts may simply decrease the prevalence of one disease at the expense of an increased prevalence of another.

## 12. The Required Approach

Clinical trials are urgently needed to conclusively test a variety of questions. The approach needs to be extensive and systematic rather than timid and piecemeal. The tests required will be on a large scale, although this scale pales in comparison to the current amount of effort and energy being poured into research on allergic, inflammatory, and autoimmune issues. This approach to medicine will not likely be supported by any committee of experts containing members with vested interests in traditional immunological approaches (either in humans or in rodents) or in the pharmaceutical industry. Rather, this approach is much more likely to be appreciated by those individuals with a broad understanding of biology and whose primary interest is in improving health care and, ultimately, the health of the population as a whole. With that in mind, the following questions need to be answered.

- Can autism be cured or effectively treated with biome reconstitution, or only prevented by biome reconstitution?
- How important for children is the biome of the mother prior to and during pregnancy, and while breastfeeding? (Does prevention require reconstitution of the mother's biome?)
- Which helminths or combination of helminths is both safe and effective? Although some concerns dealing with the use of helminths for therapy have been expressed, these objections are easily dealt with [1]. Several candidate species are well adapted to humans, asymptomatic in low numbers, and thus are obvious candidates for initial and immediate testing. Further, new technologies might be developed which could improve on species that are naturally occurring. For example, irradiation of organisms to achieve sterility and eliminate the possibility of transmission, or cultivation of human-specific helminths in ultraclean and immunodeficient rodents or even *in vitro* might improve the utility of helminths for widespread medical use.
- What medical conditions such as a suppressed immune system, anemia, or coagulopathy might be contraindications for biome reconstitution?
- Does optimal biome reconstitution vary with human genotype or other factors such as age, gender, and body size/composition?
- In addition to the restoration of helminths to the biome, what steps should be taken for restoration and maintenance of the microbiome?
- How will a reconstituted biome affect other areas of medicine, including aging, immunosuppression, infectious disease, cancer biology, and vaccine technology?

## 13. Is Autism a Postindustrial, Hyperimmune-Associated Epidemic?

The idea that autism is a result of evolutionary mismatches is predicated on the idea that autism is epidemic in postindustrial society. Thus, if the incidence of autism is the same in preindustrial societies as it is in postindustrial societies, then biome depletion is not a factor in the pathogenesis of autism, and biome reconstitution will not affect the incidence of autism. The epidemiology of autism has been hotly debated. Some changes in diagnostic criteria and awareness of autism have certainly affected changes in the reported incidence of autism over time. However, debates regarding the changing diagnosis of autism over the past few decades have little bearing on the idea that biome depletion profoundly influences autism. Rather, the pertinent question is whether the prevalence of autism has changed over the past 150 years as various cultures have transitioned from pre to postindustrial over the course of several generations. Although it may be very difficult if not impossible to estimate the prevalence of autism prior to the industrial revolution in countries that are currently postindustrial, it might still be possible to examine the prevalence of autism in some preindustrial societies. One very recent study by Schieve et al. is particularly informative in this regard: using a phone survey technique to obtain data on 4,690 Hispanic children, a “striking heterogeneity” in the prevalence of autism spectrum disorder between US-born Hispanic children with 2 US-born parents (autism spectrum disorder prevalence 2.39%) and otherwise similar children with 2 foreign-born parents (autism spectrum disorder prevalence 0.31%; *P* = 0.05) was observed [86]. This observation is nicely predicted by the idea that autism is a consequence of evolutionary mismatches in postindustrial society and is consistent with the idea that the parental immune status is important in the development of autism. Further assessment of autism in preindustrial cultures may, however, prove unnecessary; the easiest way to evaluate the idea that autism is related to biome depletion is probably to conduct the experiment. Whether prophylactic biome reconstitution does or does not affect the incidence of autism will answer all questions conclusively, just as the effects of biome reconstitution [49, 87] and vitamin D supplementation [88] on multiple sclerosis, if confirmed by future studies, will effectively end long-standing debates about the etiology of that disease.

The idea that autism is a result of evolutionary mismatches is predicated on the idea that autism often occurs in genetically “normal” individuals. Thus, if autism can be attributed to genetic factors which dictate the presence of disease even in individuals living in a preindustrial society, then biome depletion is probably not a factor in the pathogenesis of autism, and biome reconstitution will probably not affect the incidence of autism. Certainly there are cases where autism is associated with genetic mutations which are deleterious, such as fragile X syndrome, but at present most individuals with autism appear genetically normal [89]. However, it remains unknown what percentage of autism can be attributed directly to genetics, and the idea that biome depletion and other evolutionary mismatches affect mutation rates is speculative and unexplored. Again, the most effective approach to resolving the debate regarding the role of genetics may be to prophylactically reconstitute the biome of a sample population and await the results.

The epidemiology and the genetics of autism are inextricably woven together. It is anticipated that one of two pictures will emerge. Either a postindustrial epidemic of autism with normal genetics will be identified, or autism will be eventually defined as a “normal” part of the human condition, potentially associated with certain genes or combinations of genes that predispose to disease regardless of the environment. In the former view, autism is due to an evolutionary mismatch, whereas in the latter view, autism is due to evolution itself. It is of critical importance not to accept the latter view prematurely since such acceptance may disable efforts at compensating for evolutionary mismatches or discourage work on identifying postindustrial triggers for disease, with potentially tragic consequences. Fortunately, several lines of evidence argue against the idea that autism is a “normal” part of human biology. First, a general consideration of the nature of human evolution argues that evolution is not responsible for autism. The social interactions disrupted by autism are fundamental to survival of not only humans, but also a wide range of mammalian and nonmammalian species, pointing to the ancient evolutionary origins of those interactions. Given the intense selection pressures on early human populations, it seems unlikely that an error rate of 1% in social interactions would have developed and then persisted for many tens of thousands of years. However, the idea that autism was tolerated during human evolution because it is essentially a tradeoff, an inherent “side-effect” of human brain function that is required for survival, cannot be ruled out. A second line of evidence pointing in favor of autism as a result of evolutionary mismatch rather than evolution is the study, described above, showing that parental birth in a postindustrial culture seems to predispose offspring to autism [86]. Third, the observations (a) that the immune system is profoundly destabilized by postindustrial culture and (b) that brain development is closely tied with immune function, when considered together, argue strongly for the idea that it is an evolutionary mismatch, not evolution, which is responsible for autism. Indeed, to the extent that human brain development is strongly tied to the immune system, it would be difficult to explain the idea that postindustrial culture and the resulting consequences of evolutionary mismatches such as biome depletion do not profoundly affect brain development. Finally, the strong association of autism with autoantibody production [3] and other immune abnormalities points toward the etiology of autism as straight forward: another epidemic in the long list of hyperimmune associated epidemics caused by biome depletion in postindustrial society. The fact that this particular disease affects primarily the brain rather than other organs or tissues undoubtedly leads to the tremendous complexity in the pathology associated with autism, but this complexity should not blind the medical community to the simple roots that apparently underlie epidemics of all allergic and autoimmune diseases, or to the apparent solutions that can currently be implemented to prevent those diseases [1].

## 14. Summary/Conclusions

Autism has many of the characteristic features of hyperimmune-associated diseases which result from biome depletion and other consequences of biology/cultural mismatches in postindustrial culture, potentially sharing a common cause and potentially a common solution with those diseases. The identification of triggers for autism (e.g., association with viral infection during pregnancy [90]) parallels that of other diseases in this family. However, the trigger that causes disease is often unrelated to the cultural mismatches with human biology which destabilize the human biome and make the trigger dangerous. The association of autism with genetic and probably epigenetic factors also parallels that of other hyperimmune-associated diseases, but offers little in the hope of prevention, treatment, or a cure.

The ecosystem of the human body, the human biome, is a tightly interwoven collection of factors which includes immunity, brain function, sunlight, hormones, metabolism, transport of metabolites, the microbiome, and helminths. It stands firmly to reason that if one or more of these components are profoundly altered or even deleted, the entire system may become destabilized. This view of ecosystems has long been appreciated by ecologists [91]. Although some hyperimmune diseases might be eliminated if the respective triggers for those diseases are identified and avoided, such as approach cannot be successful in eliminating the overall problem; roughly 50% of children in postindustrial society have some form of chronic health condition [92], many millions are affected by a wide range of allergic, inflammatory, and autoimmune disorders, and inflammation as a result of biome destabilization may play a key role in such common maladies as heart disease and dementia. With that in mind, the need for normalization of the biome cannot be overemphasized, regardless of particular diseases that might be prevented by identifying and avoiding the environmental trigger. Thus, while it is hoped that a trigger that causes autism might be confidently identified in the near future, and new cases of autism might be greatly reduced as a result, it is hypothesized that normalization of the human biome will be necessary to avoid the full milieu of chronic immune-related diseases that plague postindustrial society.
